# Structural and Functional Insights into Foamy Viral Integrase

**DOI:** 10.3390/v5071850

**Published:** 2013-07-18

**Authors:** Md. Alamgir Hossain, Md. Khadem Ali, Cha-Gyun Shin

**Affiliations:** Department of Biotechnology, Chung-Ang University, Ansung 456-756, South Korea; E-Mails: alamgir_bge@yahoo.com (M.A.H.); khadem_bge05@yahoo.com (M.K.A.)

**Keywords:** foamy virus, integrase, anti-retroviral drugs, cellular trafficking

## Abstract

Successful integration of retroviral DNA into the host chromosome is an essential step for viral replication. The process is mediated by virally encoded integrase (IN) and orchestrated by 3'-end processing and the strand transfer reaction. *In vitro* reaction conditions, such as substrate specificity, cofactor usage, and cellular binding partners for such reactions by the three distinct domains of prototype foamy viral integrase (PFV-IN) have been described well in several reports. Recent studies on the three‑dimensional structure of the interacting complexes between PFV-IN and DNA, cofactors, binding partners, or inhibitors have explored the mechanistic details of such interactions and shown its utilization as an important target to develop anti-retroviral drugs. The presence of a potent, non-transferable nuclear localization signal in the PFV C-terminal domain extends its use as a model for investigating cellular trafficking of large molecular complexes through the nuclear pore complex and also to identify novel cellular targets for such trafficking. This review focuses on recent advancements in the structural analysis and *in vitro* functional aspects of PFV-IN.

## 1. Introduction

Integration of the linear viral cDNA into the host cell chromosome occurs through two sequential catalytic events orchestrated by the virally encoded enzyme integrase (IN) and is considered a key step in the retroviral lifecycle [1,2]. IN recognizes attachment sites at long terminal repeats on both termini of the cDNA and catalyzes two endonucleolytic reactions: (i) a 3'-processing reaction, where IN removes two terminal nucleotides from one or both 3' ends of the long terminal repeats (LTRs), adjacent to conserved CA bases and exposes reactive adenosine 3'-OH groups, and (ii) the strand transfer reaction, where IN inserts viral cDNA into the chromosomal DNA by recognizing a suitable target site on chromatin, cleaving the phosphodiester bonds on opposing strands of the target DNA, and joining the viral cDNA [3,4]. An additional reaction catalyzed by retroviral IN *in vitro* is called disintegration, which is the reverse process of strand transfer where the inserted viral DNA cleaves off from the target DNA [5]. However the *in vivo* relevance of disintegration activity on viral replication is still unknown [6].

Retroviral IN proteins are comprised of the N-terminal domain (NTD), the central catalytic core domain (CCD), and the C-terminal domain (CTD) with varying evolutionary conservation [6]. The highly conserved NTD and CCD possess metal ion binding sites. The NTD spans the first 50–80 amino acids and contains a characteristic zinc-binding HHCC motif. Approximately 150–200 amino acids are contained in the CCD, which harbors three conserved acidic residues such as Asp, Asp_35_, Glu, known as the D, D_35_, E motif, essential for catalysis of 3' processing, DNA strand transfer [7,8], and disintegration activities [9,10]. A divalent metal co-factor is required for functioning [8]. The 80–100 amino acids CTD of retroviral integrases are less highly conserved and show structural similarities with enzymes from the nuclease and polynucleotidyltransferase superfamily [11,12,13]. They also contain a cluster of conserved amino acids that serves as a binding site for two divalent cations Mn^2+^ or Mg^2+^ [7,14].

Foamy viruses (FVs) are classified into the subfamily Spumaretrovirinae based upon their differences from orthoretroviruses [15]. The initiation of DNA synthesis in the virus producing cells makes them distinct from other retroviruses [16]. Based upon this and other properties, FVs are considered a link between retroviruses and hepadnaviruses [17]. FVs are widespread in non-human primates, cats, cows, horses, and can occur in humans, by cross-species infections from non-human primates [18,19]. Although non-pathogenic to their hosts, varying cytopathic effects are observed upon infection of epithelial cells, fibroblasts, and even lymphoid-originating cells under *in vitro* culture conditions [20,21]. 

The ability of FVs to infect and integrate into the genome of a wide range of cells leads to the construction of a number of gene therapy vectors [22]. The non-pathogenicity of FVs and the higher transduction efficiency of foamy virus derived vectors are believed to make them superior to orthoretroviral vectors (reviewed in [23]). Despite having only 15% sequence similarity with HIV-1 IN, PFV IN shows similar activities and, thus, is considered a model system to analyze the mechanism of IN action and the three-dimensional views of inhibitors and HIV-1 IN interactions [24,25]. Therefore, the views obtained from such approaches could suggest and design new anti-HIV drugs for anti-HIV therapy. The higher solubility of FV IN in a variety of solutions, faster kinetic properties, and the broad range of substrates utilized makes it easier to study [4,26]. 

## 2. Genomic and Structural Basis of FV Integration

Generally, retroviral *pol* genes are expressed as Gag-Pol fusion proteins [27]. However, FV *pol* genes are expressed from a spliced mRNA separately from *gag* [28,29,30]. FV mutants expressing Pol as a Gag-Pol fusion protein are capable of performing all of its enzymatic activities but lead to the production of non-infectious viruses [31,32,33]. Experimental evidence suggests two alternative models for Pol encapsidation into virions; however, the exact mechanism remains elusive [34,35,36].

Autocatalytic cleavage of the Pol precursor protein of other retroviruses leads to the production of protease (PR), reverse transcriptase (RT) and RNase H (RN), and IN, whereas a single cleavage event in FVs causes cleavage of the 127 kDa Pol protein into two nuclear proteins, an approximately 85 kDa protein containing the PR, RT, and RN domains and a 40-kDa protein consisting of only the IN domain [37,38]. 

Among the three domains of retroviral IN proteins the structure of the NTD in solution reveals a compact three-helical bundle in which Zn^2+^ coordination occurs through His and Cys residues of the invariant HHCC motif [39,40]. Similar to retrotransposons and some bacterial transposases, retroviral IN CCD domains harbor a highly conserved D, D_35_, E amino acid sequence motif [8,9], and the invariant D and E residues (D_64_, D_116_, and E_152_ for HIV-1) are responsible for catalysis of all enzymatic activities such as 3' processing, DNA strand transfer [8,41] and disintegration [9,10]. The CTD is rich in K and R residues and has non-specific DNA-binding activity [42,43,44].

Protein mixing experiments have revealed similar IN domain organizations from *Gammaretrovirus* (murine leukemia virus and the feline leukemia virus) [45] and *Spumavirus* [46]. PFV IN is enzymatically active *in vitro* and is homologous to other retroviral INs [47,48]. Although these three retroviral IN domains comprise ~290 amino acid residues linked through inter-linkers, PFV IN is significantly longer and comprises about 392 residues [26,49]. The possibility exists that an additional domain consisting of approximately 50 residues might be present at the N-terminus, preceding the NTD domain. Crystallographic analysis of PFV IN and DNA complexes has revealed an extra domain termed the N-terminal extension domain (NED) [25], which has no role in *in vitro* activity [24] however is essential in complexing with viral DNA (vDNA) [6].

Although numerous efforts have focused on structural analysis of HIV IN to develop new antiviral compounds and to unveil the enzymatic mechanisms, the exact three-dimensional structure of the full protein or its functional mechanism is still unknown [50,51]. The requirements of high concentration [52] of long viral DNA substrates [53,54,55] and LEDGF (lens epithelium derived growth factor) complexed IN made the X-ray crystallographic analysis of the HIV-1 intasome a difficult task. In contrast, remarkable solubility, utilization of exclusively short viral DNA substrates [24] for concerted integration assays made the IN from PFV an excellent ortholog of HIV-IN for *in vitro* experimentation and crystallographic determination of the retroviral intasome. The crystal structure of the PFV intasome provided insight into the HIV-1 drug resistance mechanism, as it has a 22% amino acid sequence similarity within the active sites of CCD domains of HIV-1 [25,56].

Symmetric intasome complexes assembled with full-length PFV IN, Zn^2+^, and pre-processed U5 viral DNA substrate retained concerted integration activity [25]. Intasome crystals differ in the basic Zn^2+^-IN-vDNA assembly and the presence of ligands: Mn^2+^ or Mg^2+^, target DNA, or inhibitors were yielded from the PFV system [25]. The intasome structure is composed of a dimer of IN dimers and a pair of synapsed vDNA ends. The pairs of IN subunits are functionally and structurally distinct as predicted by partial structures [57,58]. The inner subunits adopt an extended conformation and are responsible for all interactions with viral DNA and catalysis. The outer IN subunits do not interact with each other or the viral DNA and seem to play a supporting role. Interaction between molecules within each dimer occurred through an extensive CCD-CCD interface (reviewed in [59]). However the NTD and CTD domains of the outer subunits are disordered in the crystals and their functions are currently unknown [6,60]. Soaking PFV intasome crystals in MnCl_2_ revealed metal bound intasomal active sites [25] that support the previous prediction about the presence of two metal ions at each of the active sites [61].

## 3. Biochemical and Enzymatic Properties of FV Integrase

The function of IN is to insert viral DNA into the host genome, and the reaction is accomplished by 3'-processing and DNA strand transfer steps, which are bimolecular nucleophilic substitution (S_N_^2^) reactions. Both steps are simultaneously accomplished by metal-dependent nucleotidyl transferase and nuclease action, like transposases and RNase H enzymes and both are catalyzed by a triad of acidic residues in a characteristic D, D_35_, E motif found in all retroviral INs [62,63,64]. The catalytic site residues coordinate the positions of two Mg^2+^ ions to activate the attacking nucleophile (the oxygen atom of a water molecule for 3'-processing and the 3'-hydroxyl of viral DNA for strand transfer) and destabilize the scissile phosphodiester bonds [65,66,67]. IN removes the terminal dinucleotides (GT in HIV-1, TT in ASLV avian sarcoma leukosis virus) from each 3'-end of the double-stranded viral DNA during the processing step. However, FV integration does not involve the removal of two terminal nucleotides from its U3 region. In contrast, two terminal nucleotides (AT) are removed from its U5 region in order to provide the subterminal CA for the joining reaction to host cell DNA [68]. In the second step, a nucleophile attacks the free 3'-hydroxyl of the viral DNA on the target chromosomal DNA, resulting in covalent joining of the two molecules. The subsequent removal of the two unpaired nucleotides at each 5'-overhanging end of the viral DNA and filling of the gaps are most likely performed by host enzymes. 

Although HIV-1 IN has long been studied to develop inhibitors, the three-dimensional characterization of intact HIV-1 IN structure is unknown because of its poor solubility. This problem has been tried to be overcome by studying individual domains and introducing a mutation into the catalytic domain [69]. Studies over the past decade have determined the structure of all three HIV-1 IN domains [12,39,40,44]. Structural characterization of the intasome, which comprises the IN, viral DNA, and associated host cellular proteins, will help gain insight into inhibitor function. The first retroviral intasome that has been successfully characterized is the PFV intasome [25].

Several detergents are generally used for improving the solubility of recombinant IN and these might have effects on the *in vitro* enzymatic actvities of INs. We investigated the effect of various chemicals on feline foamy viral integrase (FFV IN) enzymatic activities [26]. We evaluated the potential effects of CHAPS, glycerol, Tween 20, and Triton X-100 on 3'-processing activities. Although glycerol and Triton X-100 had no inhibitory activity up to a 50% final concentration, the non-ionic detergent showed inhibitory effects at 10 mM. However, no effect of Tween 20 was observed at a high concentration due to its viscous nature. 

The isolated CCD domain from HIV-1 [70] and ASLV [71] IN proteins failed to execute 3' processing and DNA strand transfer activities. Evidence from several studies indicates that a tetrameric form of the retroviral IN is required for concerted *in vitro* integration [72,73]. Mixtures of HIV-1 IN with defective NTD and CTD deletion mutants support 3' processing and DNA strand transfer activities, suggesting that these enzymes function as multimers [74,75]. HIV-1 IN exists in tetramer-dimer equilibrium, with the tetrameric form predominating at concentrations as low as 0.2 mM, but PFV IN mostly exists as a monomer at concentrations <30 mM [4,76]. Size exclusion chromatography has failed to reveal the multimeric forms of PFV IN [24].

PFV IN shows efficient catalytic properties that make it a more elegant IN to study different enzymatic activities than HIV IN [77]. A very large excess of enzyme compared with DNA is required (usually >30:1) to accomplish 3'-processing activity by HIV-1 IN, which hampers study of its catalytic properties. PFV IN forms a relatively stronger complex with synthetic DNA duplexes that imitates the terminal sequence of the viral DNA long terminal repeat U5 domain [78]. Fluorescence polarization of the DNA-binding kinetics at 25 °C revealed that DNA-HIV IN forms in 3–4 minutes, which is approximately five times longer than the time required for DNA association with PFV IN [78]. This observation indicates the greater favorability of PFV IN binding to DNA [77].

## 4. Functional Aspects: *In Vivo* and *in Vitro*

Retroviral INs belong to the retroviral IN superfamily (RISF) which comprises nucleic acid metabolizing enzymes such as RNase H, RuvC (*Escherichia coli* ruvC gene derived endonuclease), bacteriophage MuA transposase, and nuclease component of the RISC complex Argonaute [62]. These enzymes are characterized by electronegative D and E side chains in their catalytic domains [12,64]. Observations from solution biochemistry [73,79], chemical cross-linking [72], and structurally based approaches [25,80] have revealed that an IN tetramer is required for DNA strand transfer activity and an IN multimer as well as dimer catalyzes 3' processing activity *in vitro* [57,72]*.* However, the PFV IN tetramer efficiently processes a pair of LTR ends *in crystallo* [65]. 

Recombinant PFV IN is capable of executing 3'-processing and half-site strand transfer *in vitro* [81,82]. One study found that these enzymes also catalyze concerted integration *in vitro* under stringent conditions [24]. In the case of HIV-1 IN, unprocessed DNA molecules of several hundred base pairs and an optimal concentration of DMSO and/or PEG are required for concerted integration *in vitro* [53,54]. In contrast, PFV IN is capable of utilizing preprocessed oligonucleotide donor DNA substrates as short as 16 bp to carry out almost exclusive concerted strand transfer *in vitro* and under physiological conditions [24]. Unlike lentiviral INs, only one end (U5) of the processed cDNA is utilized for processing by the *Spumavirus* IN [83].

PFV IN is highly proficient at concerted integration *in vitro* compared to that of HIV-1 IN, as it generates a considerable amount of half-site products. A higher concentration of HIV-1 IN negatively regulates the concerted integration efficiency, and the optimum concentration used is 5–15 nM [79]. These observations indicate that a lower multimeric form is required before interacting with its donor DNA substrate. However, PFV IN is less prone to form multimers, which allows for studies of the concerted retroviral integration process at higher IN and donor DNA inputs and to visualize the products [24]. Interestingly, these properties of PFV IN are extremely helpful in structural studies of the retroviral synaptic complex (IN bound long terminal repeat DNA ends). 

The LTR sequence of viral DNA is the only sequence that can be recognized by retroviral IN and is necessary to perform the enzymatic activities successfully [84,85]. We investigated substrate specificity of three retroviral INs by incubating different LTR substrates such as HIV-1, PFV, and FFV. HIV-1 IN and PFV IN utilized their own substrates highly specifically and cleaved only their own substrates, but FFV IN is non-specific and efficiently cleaves its own substrate as well as the HIV-1 LTR and PFV LTR substrates [26].

Retroviral integration is not entirely random and shows genus-specific biases on the availability of target DNA sequence in the reported genomic scales [86]. Members of the Lentivirus family including HIV-1 IN integrate along the bodies of active genes and integration negatively correlates with the uppermost levels of transcriptional activity [87] but members of the Gammaretrovirus family favor integration at the promoter sites and CpG islands [88,89,90]. However PFV showed reduced frequency of integration within transcription units and therefore showed less effects on local gene expression [91,92].

## 5. Metal Use Is Distinct among Retroviruses

Retroviral IN has two metal binding domains; the NTD containing a zinc finger motif, which binds Zn^2+^, and the CCD, containing a conserved D, D_35_, E motif, which has a tendency to bind Mn, or other metal cofactors [8,93]. Mg^2+^ is a natural cofactor due to its abundant presence *in vivo*; however, Mn^2+^ also acts as cofactor *in vitro* [76,94].

Similar to other members of the nucleotidyl transferases protein family, the IN active site is composed of acidic residues and performs enzymatic activities through a two-metal-cation mechanism and the nature and number of divalent metal cations required for catalysis have already been identified [95,96]. Processing and joining reactions are two IN enzymatic activities performed by the CCD where Mg^2+^ or Mn^2+^ cations act as co-factors *in vitro*. Some studies have successfully investigated active site complex formation using physiologically irrelevant but stronger binding metals, such as Zn^2+^, Cd^2+^, or Ca^2+^ with ASV IN [95] and Cd^2+^ with HIV-1 IN [96], however a recent study reported a PFV IN/Mg^2+^ or Mn^2+^ intasome assembly with two metal cations at the active site [25]. The reason for zinc coordination is due to its ability to accept four tetrahedrally arranged ligands. Although it supports endonucleolytic activity *in vitro*, it cannot be used as cofactor for IN catalysis *in vivo* [97].

We investigated the metal requirements for FV IN enzymatic activities by using FFV IN. Our results demonstrated that similar to other retroviral IN, FFV IN successfully utilizes Mn^2+^ or Mg^2+^ as a cofactor in three different enzymatic reactions [26]. We also screened the capacity of FFV IN to utilize other metal ions as co-factors. Besides Mn^2+^ or Mg^2+^, Co^2+^ and Zn^2+^ ions act as cofactors for 3'‑processing activity of FFV IN, as they induce enzymatic activity in the absence of the Mn^2+^ ion and show concentration-dependent induction [26]. 

## 6. Structural Basis of IN Inhibitor Action

Zidovudine (AZT) was the first antiretroviral agent introduced in 1986 [98] and utilized as therapeutic agent in antiretroviral therapy [99]. A total of 26 antiretroviral compounds classified into six classes (nucleoside/nucleotide reverse transcriptase inhibitors, N(t)RTIs; non-nucleoside reverse transcriptase inhibitors, NNRTIs; protease inhibitors, PIs; fusion inhibitors; co-receptor blockers and integrase strand transfer inhibitors, INSTIs) are currently approved for clinical use [100]. Raltegravir is an INSTI, causes a strong inhibition of integration, and reduces viral load [101]. Elvitegravir is a Phase III INSTI and has been approved for HAART by the FDA [102,103,104]. Infectivity of PFV in cell culture and *in vitro* enzymatic activities of PFV IN showed a significant sensitivity to raltgravir and elvitegravir [24]. The high degree of amino acid sequence similarity within the active sites of HIV-1 and PFV INs and superposition of active side residues in crystals [24] implies the possibility of HIV-1 IN strand transfer inhibitors to be equally active against PFV IN [24,105,106]. Soaking PFV intasome crystals in raltegravir or other INSTIs has revealed that these compounds bind the active site and are interacted by their oxygen atoms with bound metal ions at the IN active site [25]. Crystallographic analysis of PFV intasome and INSTIs interactions explained the divergent binding mechanisms within the active site and hence explore the possibility to determine the raltegravir-resistance mutations [25].

## 7. NLS of FV Integrase

Successful infection and replication of retroviruses in their host depend largely on the reverse transcription of their RNA genome to cDNA and integration into the host cell chromosome (reviewed in [81]). Integration occurs in the nucleus; therefore, viral cDNA complexed with other viral and cellular proteins forms a large nucleoprotein complex termed the preintegration complex (PIC), which translocates from the cytoplasm to the nucleus [107]. Various cellular nuclear-import receptors as well as viral karyophilic proteins have been suggested to be involved for the successful translocation of PIC into the nucleus. Among the cellular nuclear-import receptors involvment of the importin a/ß heterodimer [108,109,110], importin 7 [111,112] and transportin 3 (TPNO3/transportin-SR2) [113,114,115] have been extensively studied. Another cellular protein, lens epithelium-derived growth factor/p75 (LEDGF/p75) plays a vital role in HIV-1 PIC translocation into the nuclei of infected cells [116,117,118]. TNPO3/ Transportin-SR2 is a member of the importin ß family, which recognizes serine–arginine-rich repeats within precursor-mRNA splicing factors and subsequently transports these factors into the nucleus [119,120] suggesting the possible role of TNPO3/Transportin-SR2 in transporting the HIV-1 PIC into the nucleus. Moreover RNAi-mediated knockdown of TNPO3/Transportin-SR2 causes the inhibition of HIV-1 replication, after reverse transcription and before the covalent attachment of viral cDNA to host chromosomal DNA [113]. This findings imply that TNPO3/Transportin-SR2 is either necessary for viral cDNA transportation or optimal integration activity [114]. Among the suggested karyophilic viral proteins, such as the Matrix (MA), Vpr and IN, integrase remains a reasonable candidate for a functionally relevant target of nuclear import factors as it plays an essential role in the nucleus during the early steps of infection [114]. Screening of cellular proteins for possible binding partners of IN, revealed TNPO3/Transportin-SR2 as an integrase binding protein. Moreover a recent study based on co-immunoprecipitation (co-IP) and immunostaining experiments and the use of cellpermeable functional peptides suggested that in addition to importin α, TNPO3 is involved in nuclear import of IN in virus-infected cells [121]. Retroviral INs have nuclear localization signals (NLS) [122] and therefore perform two important roles in the viral life cycle. They transport viral cDNA from the cytoplasm to the nucleus and integrate viral cDNA into the cellular genomic DNA [62,84]. Members of the retroviral family possess NLS at different IN residues. HIV-1 IN possesses a basic bipartite type NLS at residues 186–188 and 211–219 [123]. An additional NLS in the central domain of the HIV-1 IN has been reported at residues 161–173 [124]. In the feline immunodeficiency virus IN, the karyophilic determinant has been mapped to the highly conserved N‑terminal zinc binding HHCC motif. The karyophilic determinant for ASLV IN has been proposed to be a noncanonical NLS that maps to six basic amino acids located within residues 207–235 of the protein [125]. Besides IN, other viral proteins with an NLS sequence include the matrix (MA) domain of Gag and Vpr of lentiviruses, which are thought to be important for importing PIC into the nuclei of growth-arrested cells [126,127,128].

Several studies have indicated that FV IN contains NLS sequences [129,130]. A monoclonal antibody targeted to FV IN suggests that the IN domain of the Pol protein has a NLS responsible for import into the nucleus [35]. It has been suggested that as a part of PFV PIC, the Gag protein translocates to the nucleus and facilitates integration by tethering the viral DNA genome to the host cell chromatin [131], but a separate study showed that translocation of Gag and the viral genome to the nucleus is dependent on IN in both cycling and growth-arrested cells [132]. It has also been shown that the FV genome and Gag can enter the nuclei of growth-arrested cells, indicating the ability of the FV PIC to cross an intact nuclear membrane similar to that of lentiviruses [132]. However, cells transduced with IN-deficient vectors reveal no Gag or viral genome within nuclei, suggesting that Gag alone is not critical for PIC transport to the nucleus, whereas IN is an absolute requirement [132]. This study did not find any dependence of Gag nuclear localization on IN in cycling cells. Therefore, transport of Gag to the nucleus is totally independent of IN [132]. 

We investigated the karyophilic determinant of PFV IN, and our results demonstrated that PFV IN possesses a potent NLS in its C-terminal domain spanning from residues 289–371 [129]. Studies on seven point mutants created at that region by changing the basic amino acids lysine or arginine to threonine or proline revealed that a mutation at positions 305 and 315 did not affect nuclear localization of PFV-IN but mutations at positions 308, 313, 318, 324, and 329, significantly altered nuclear localization. Further observations indicate that PFV-IN NLS is non-transferable and different from classical NLS, which has five discontinuous basic amino acids. 

## 8. Conclusion

Although much effort has long been undertaken to use HIV-1 IN as a target for anti-retroviral drug development, the exact structural elucidation of full-length protein remains unknown. Rapid emergence of HIV-1 IN inhibitor resistant strains necessitate the knowledge of the structural details of active site geometry and IN domain interactions in order to develop more effective drugs. PFV IN is equally sensitive to HIV-1 IN inhibitors therefore the crystal structures of PFV intasome as the only determined retroviral intasome will help to elucidate the structural basis of HIV-1 drug resistance to clinical INSTIs. This review provides insights about the biochemical and enzymatic activities of FV IN and the reasons for using FV IN as a model system to investigate the structural basis of anti-retroviral therapy as well as to improve the safety of FV vector systems. 

## References

[1] Craigie R., Craig N.L., Craigie R., Gellert M., Lambowitz A.M. (2002). Retroviral DNA integration. Mobile DNA II.

[2] Lewinski M.K., Bushman F.D. (2005). Retroviral DNA integration—Mechanism and consequences. Adv. Genet..

[3] Ciuffi A., Bushman F.D. (2006). Retroviral DNA integration: HIV and the role of LEDGF/p75. Trends Genet..

[4] Delelis O., Carayon K., Guiot E., Leh H., Tauc P., Brochon J.C., Mouscadet J.F., Deprez E. (2008). Insight into the integrase-DNA recognition mechanism. J. Biol. Chem..

[5] Chow S.A., Vincent K.A., Ellison V., Brown P.O. (1992). Reversal of integration and DNA splicing mediated by integrase of human immunodeficiency virus. Science.

[6] Li X., Krishnan L., Cherepanov P., Engelman A. (2011). Structural biology of retroviral DNA integration. Virology.

[7] Delelis O., Carayon K., Guiot E., Leh H., Tauc P., Brochon J.C., Mouscadet J.F., Deprez E. (2008). Insight into the integrase -DNA recognition mechanism. J. Biol. Chem..

[8] Kulkosky J., Jones K.S., Katz R.A., Mack J.P., Skalka A.M. (1992). Residues critical for retroviral integrative recombination in a region that is highly conserved among retroviral/retrotransposon integrases and bacterial insertion sequence transposases. Mol. Cell. Biol..

[9] Engelman A., Craigie R. (1992). Identification of conserved amino acid residues critical for human immunodeficiency virus type 1 integrase function *in vitro*. J. Virol..

[10] Leavitt A.D., Shiue L., Varmus H.E. (1993). Site-directed mutagenesis of HIV-1 integrase demonstrates differential effects on integrase functions *in vitro*. J. Biol. Chem..

[11] Bujacz G., Alexandratos J., Zhou-Liu Q., Clement-Mella C., Wlodawer A. (1996). The catalytic domain of human immunodeficiency virus integrase: Ordered active site in the F185H mutant. FEBS Lett..

[12] Dyda F., Hickman A.B., Jenkins T.M., Engelman A., Craigie R., Davies D.R. (1994). Crystal structure of the catalytic domain of HIV-1 integrase: Similarity to other polynucleotidyl transferases. Science.

[13] Davies J.F., Hostomska Z., Hostomsky Z., Jordan S.R., Matthews D.A. (1991). Crystal structure of the ribonuclease H domain of HIV-1 reverse transcriptase. Science.

[14] Khan E., Mack J.P., Katz R.A., Kulkosky J., Skalka A.M. (1991). Retroviral integrase domains: DNA binding and the recognition of LTR sequences. Nucleic Acids Res..

[15] Fauquet C.M., Mayo M.A., Maniloff J., Desselberger U., Ball L.A. (2005). Virus taxonomy. Eighth Report of the International Committee on Taxonomy of Viruses.

[16] Yu S.F., Baldwin D.N., Gwynn S.R., Yendapalli S., Linial M.L. (1996). Human foamy virus replication: A pathway distinct from that of retroviruses and hepadnaviruses. Science.

[17] Linial M.L. (1999). Foamy viruses are unconventional retroviruses. J. Virol..

[18] Lacellier C.-H., Saib A. (2000). Minireview—Foamy viruses: Between retroviruses and pararetroviruses. J. Virol..

[19] Epstein M.A. (2004). Simian retroviral infections in human beings. Lancet.

[20] Mergia A., Leung N.J., Blackwell J. (1996). Cell tropism of the simian foamy virus type 1 (SFV-1). J. Med. Primatol..

[21] Mikovits J.A., Hoffman P.M., Rethwilm A., Ruscetti F.W. (1996). *In vitro* infection of primary and retrovirus-infected human leukocytes by human foamy virus. J. Virol..

[22] Leurs C., Jansen M., Pollok K.E., Heinkelein M., Schmidt M., Wissler M., Lindemann D., von Kalle C., Rethwilm A., Williams D.A. (2003). Comparison of three retroviral vector systems for transduction of nonobese diabetic/severe combined immunodeficiency mice repopulating human CD34+ cord blood cells. Hum. Gene. Ther..

[23] Lindemann D., Rethwilm A. (2011). Foamy virus biology and its application for vector development. Viruses.

[24] Valkov E., Gupta S.S., Hare S., Helander A., Roversi P., McClure M., Cherepanov P. (2009). Functional and structural characterization of the integrase from the prototype foamy virus. Nucleic Acids Res..

[25] Hare S., Gupta S.S., Valkov E., Engelman A., Cherepanov P. (2010). Retroviral intasome assembly and inhibition of DNA strand transfer. Nature.

[26] Hyun L.D., Hyun U., Kim J.Y., Shin C.-G. (2010). Characterization of biochemical properties of feline foamy virus integrase. J. Microbiol. Biotechnol..

[27] Linial M, Fan H., Hahn B., Löwer R., Neil J., Quackenbusch S., Rethwilm A., Sonigo P., Stoye J., Tristem M., Fauquet C.M., Mayo M.A., Maniloff J., Desselberger U., Ball L.A. (2005). Retroviridae. Virus Taxonomy.

[28] Yu S.F., Edelmann K., Strong R.K., Moebes A., Rethwilm A., Linial M.L. (1996). The carboxyl terminus of the human foamy virus Gag protein contains separable nucleic acid binding and nuclear transport domains. J. Virol..

[29] Bodem J., Löchelt M., Winkler I., Flower R.P., Delius H., Flügel R.M. (1996). Characterization of the spliced pol transcript of feline foamy virus: The splice acceptor site of the pol transcript is located in gag of foamy viruses. J. Virol..

[30] Jordan I., Enssle J., Güttler E., Mauer B., Rethwilm A. (1996). Expression of human foamy virus reverse transcriptase involves a spliced pol mRNA. Virology.

[31] Swiersy A., Wiek C., Reh J., Zentgraf H., Lindemann D. (2011). Orthoretroviral-like prototype foamy virus gag-pol expression is compatible with viral replication. Retrovirology.

[32] Lee E.G., Sinicrope A., Jackson D.L., Yu S.F., Linial M.L. (2012). Foamy Virus Pol Protein Expressed as a Gag-Pol Fusion Retains Enzymatic Activities, Allowing for Infectious Virus Production. J. Virol..

[33] Jackson D.L., Lee E.G., Linial M.L. (2012). Expression of prototype foamy virus pol as a gag-pol fusion protein does not change the timing of reverse transcription. J. Virol..

[34] Lee E.G., Linial M.L. (2008). The C terminus of foamy retrovirus Gag contains determinants for encapsidation of Pol protein into virions. J. Virol..

[35] Heinkelein M., Leurs C., Rammling M., Peters K., Hanenberg H., Rethwilm A. (2002). Pregenomic RNA is required for efficient incorporation of pol polyprotein into foamy virus capsids. J. Virol..

[36] Peters K., Wiktorowicz T., Heinkelein M., Rethwilm A. (2005). RNA and protein requirements for incorporation of the Pol protein into foamy virus particles. J. Virol..

[37] Netzer K.O., Schliephake A., Maurer B., Watanabe R., Aguzzi A., Rethwilm A. (1993). Identification of pol-related gene products of human foamy virus. Virol.

[38] Pfrepper K.I., Rackwitz H.R., Schnolzer M., Heid H., Löchelt M., Flügel R.M. (1998). Molecular characterization of proteolytic processing of the Pol proteins of human foamy virus reveals novel features of the viral protease. J. Virol..

[39] Cai M., Zheng R., Caffrey M., Craigie R., Clore G.M., Gronenborn A.M. (1997). Solution structure of the N-terminal zinc binding domain of HIV-1 integrase. Nat. Struct. Biol..

[40] Eijkelenboom A.P., van den Ent F.M., Vos A., Doreleijers J.F., Hard K., Tullius T.D., Plasterk R.H., Kaptein R., Boelens R. (1997). The solution structure of the amino-terminal HHCC domain of HIV-2 integrase: A three-helix bundle stabilized by zinc. Curr. Biol..

[41] Drelich M., Wilhelm R., Mous J. (1992). Identification of amino acid residues critical for endonuclease and integration activities of HIV-1 IN protein *in vitro*. Virology.

[42] Vink C., Oude Groeneger A.M., Plasterk R.H. (1993). Identification of the catalytic and DNA-binding region of the human immunodeficiency virus type I integrase protein. Nucleic Acids Res..

[43] Engelman A., Hickman A.B., Craigie R. (1994). The core and carboxyl-terminal domains of the integrase protein of human immunodeficiency virus type 1 each contribute to nonspecific DNA binding. J. Virol..

[44] Lodi P.J., Ernst J.A., Kuszewski J., Hickman A.B., Engelman A., Craigie R., Clore G.M., Gronenborn A.M. (1995). Solution structure of the DNA binding domain of HIV-1 integrase. Biochemistry.

[45] Jonsson C., Donzella G., Gaucan E., Smith C., Roth M. (1996). Functional domains of Moloney murine leukemia virus integrase defined by mutation and complementation analysis. J. Virol..

[46] Pahl A., Flugel R.M. (1995). Characterization of the human spuma retrovirus integrase by site-directed mutagenesis, by complementation analysis, and by swapping the zinc finger domain of HIV-1. J. Biol. Chem..

[47] Dias H.W., Aboud M., Flugel R.M. (1995). Analysis of the phylogenetic placement of different spumaretroviral genes reveals complex pattern of foamy virus evolution. Virus Genes.

[48] Schweizer M., Neumann-Haefelin D. (1995). Phylogenetic analysis of primate foamy viruses by comparison of pol sequences. Virology.

[49] Bushman F.D., Wang B. (1994). Rous sarcoma virus integrase protein: Mapping functions for catalysis and substrate binding. J. Virol..

[50] Sato M., Motomura T., Aramaki H., Matsuda T., Yamashita M., Ito Y., Kawakami H., Matsuzaki Y., Watanabe W., Yamataka K. (2006). Novel HIV-1 integrase inhibitors derived from quinolone antibiotics. J. Med. Chem..

[51] Summa V., Petrocchi A., Bonelli F., Crescenzi B., Donghi M., Ferrara M., Fiore F., Gardelli C., Gonzalez Paz O., Hazuda D.J. (2008). Discovery of raltegravir, a potent, selective orally bioavailable HIV-integrase inhibitor for the treatment of HIVAIDS infection. J. Med. Chem..

[52] Hare S., Shun M.C., Gupta S.S., Valkov E., Engelman A., Cherepanov P. (2009). A novel co crystal structure affords the design of gain-of-function lentiviral integrase mutants in the presence of modified PSIP1/LEDGF/p75. PLoS Pathog..

[53] Sinha S., Grandgenett D.P. (2005). Recombinant human immunodeficiency virus type 1 integrase exhibits a capacity for full-site integration *in vitro* that is comparable to that of purified preintegration complexes from virus-infected cells. J. Virol..

[54] Li M., Craigie R. (2005). Processing of viral DNA ends channels the HIV-1 integration reaction to concerted integration. J. Biol. Chem..

[55] Sinha S., Pursley M.H., Grandgenett D.P. (2002). Efficient concerted integration by recombinant human immunodeficiency virus type 1 integrase without cellular or viral cofactors. J. Virol..

[56] Hare S., Smith S.J., Métifiot M., Jaxa-Chamiec A., Pommier Y., Hughes S.H., Cherepanov P. (2011). Structural and functional analyses of the second-generation integrase strand transfer inhibitor dolutegravir (S/GSK1349572). Mol. Pharmacol..

[57] Hare S., di Nunzio F., Labeja A., Wang J., Engelman A., Cherepanov P. (2009). Structural basis for functional tetramerization of lentiviral integrase. PLoS Pathog..

[58] Wang J.Y., Ling H., Yang W., Craigie R. (2001). Structure of a two-domain fragment of HIV-1 integrase: Implications for domain organization in the intact protein. EMBO J..

[59] Puras-Lutzke R.A., Vink C., Plasterk R.H.A. (1994). Characterization of the minimal DNA-binding domain of the HIV integrase protein. Nucleic Acids Res..

[60] Cherepanov P., Maertens G.N., Hare S. (2011). Structural insights into the retroviral DNA integration apparatus. Curr. Opin. Struct. Biol..

[61] Nowotny M., Gaidamakov S.A., Crouch R.J., Yang W. (2005). Crystal structures of RNase H bound to an RNA/DNA hybrid: Substrate specificity and metal-dependent catalysis. Cell.

[62] Engelman A., Mizuuchi K., Craigie R. (1991). HIV-1 DNA integration: Mechanism of viral DNA cleavage and DNA strand transfer. Cell.

[63] Davies D.R., Goryshin I.Y., Reznikoff W.S., Rayment I. (2000). Threedimensional structure of the Tn5 synaptic complex transposition intermediate. Science.

[64] Nowotny M. (2009). Retroviral integrase superfamily: The structural perspective. EMBO Rep..

[65] Hare S., Maertens G.N., Cherepanov P. (2012). 3'-Processing and strand transfer catalysed by retroviral integrase in crystallo. EMBO J..

[66] Mizuuchi K. (1992). Polynucleotidyl transfer reactions in transpositional DNA recombination. J. Biol. Chem..

[67] Yang W., Lee J.Y., Nowotny M. (2006). Making and breaking nucleic acids: Two Mg2+-ion catalysis and substrate specificity. Mol. Cell..

[68] Enssle J., Moebes A., Heinkelein M., Panhuysen M., Mauer B., Matthias Schweizer M., Neumann-Haefelin D., Rethwilm A. (1999). An active foamy virus integrase is required for virus replication. J. Gen. Virol..

[69] Jenkins T.M., Hickman A.B., Dyda F., Ghirlando R., Davies D.R., Craigie R. (1995). Catalytic domain of human immunodeficiency virus type 1 integrase: Identification of a soluble mutant by systematic replacement of hydrophobic residues. Proc. Natl. Acad. Sci. USA.

[70] Bushman F.D., Engelman A., Palmer I., Wingfield P., Craigie R. (1993). Domains of the integrase protein of human immunodeficiency virus type 1 responsible for polynucleotidyl transfer and zinc binding. Proc. Natl. Acad. Sci. USA.

[71] Kulkosky J., Katz R.A., Merkel G., Skalka A.M. (1995). Activities and substrate specificity of the evolutionarily conserved central domain of retroviral integrase. Virology.

[72] Faure A., Calmels C., Desjobert C., Castroviejo M., Caumont-Sarcos A., TarragoLitvak L., Litvak S., Parissi V. (2005). HIV-1 integrase crosslinked oligomers are active *in vitro*. Nucleic Acids Res..

[73] Li M., Mizuuchi M., Burke T.R., Craigie R. (2006). Retroviral DNA integration: Reaction pathway and critical intermediates. EMBO J..

[74] Ellison V., Gerton J., Vincent K.A., Brown P.O. (1995). An essential interaction between distinct domains of HIV-1 integrase mediates assembly of the active multimer. J. Biol. Chem..

[75] Engelman A., Bushman F.D., Craigie R. (1993). Identification of discrete functional domains of HIV-1 integrase and their organization within an active multimeric complex. EMBO J..

[76] Gupta K., Curtis J.E., Krueger S., Hwang Y., Cherepanov P., Bushman F.D., van Duyne G.D. (2012). Solution conformations of prototype foamy virus integrase and its stable synaptic complex with U5 viral DNA. Structure.

[77] Knyazhanskaya E.S., Smolov M.A., Kondrashina O.V., Gottikh M.B. (2009). Relative comparison of catalytic characteristics of human foamy virus and hiv-1 integrases. Acta Naturae.

[78] Smolov M., Gottikh M., Tashlitskii V., Korolev S., Demidyuk I., Brochon J.C., Mousecadet J.F., Depreze E. (2006). Kinetic study of the HIV-1 DNA 3'-end processing. FEBS J..

[79] Pandey K.K., Sinha S., Grandgenett D.P. (2007). Transcriptional coactivator LEDGF/p75 modulates human immunodeficiency virus type 1 integrase-mediated concerted integration. J. Virol..

[80] Kotova S., Li M., Dimitriadis E.K., Craigie R. (2010). Nucleoprotein intermediates in HIV-1 DNA integration visualized by atomic force microscopy. J. Mol. Biol..

[81] Pahl A., Flugel R.M. (1993). Endonucleolytic cleavages and DNA-joining activities of the integration protein of human foamy virus. J. Virol..

[82] Pahl A., Flügel R.M. (1995). Characterization of the human spuma retrovirus integrase by site directed mutagenesis, by complementation analysis, and by swapping the zinc finger domain of HIV-1. J. Biol. Chem..

[83] Juretzek T., Holm T., Gartner K., Kanzler S., Lindemann D., Herchenroder O., Picard-Maureau M., Rammling M., Heinkelein M., Rethwilm A. (2004). Foamy virus integration. J. Virol..

[84] Bushman F.D., Craigie R. (1990). Sequence requirements for integration of Moloney murine leukemia virus DNA *in vitro*. J. Virol..

[85] Roth M.J., Schwartzberg P.L., Goff S.P. (1989). Structure of the termini of DNA intermediates in the integration of retroviral DNA: Dependence on IN function and terminal DNA sequence. Cell.

[86] Bushman F., Lewinski M., Ciuffi A., Barr S., Leipzig J., Hannenhalli S., Hoffmann C. (2005). Genome-wide analysis of retroviral DNA integration. Nat. Rev. Microbiol..

[87] Schroder A.R.W., Shinn P., Chen H., Berry C., Ecker J.R., Bushman F. (2002). HIV-1 integration in the human genome favors active genes and local hotspots. Cell.

[88] Crise B., Li Y., Yuan C., Morcock D.R., Whitby D., Munroe D.J., Arthur L.O., Wu X. (2005). Simian immunodeficiency virus integration preference is similar to that of human immunodeficiency virus type 1. J. Virol..

[89] MacNeil A., Sankale J.L., Meloni S.T., Sarr A.D., Mboup S., Kanki P. (2006). Genomic sites of human immunodeficiency virus type 2 (HIV-2) integration: Similarities to HIV-1 *in vitro* and possible differences in vivo. J. Virol..

[90] Hacker C.V., Vink C.A., Wardell T.W., Lee S., Treasure P., Kingsman S.M., Mitrophanous K.A., Miskin J.E. (2006). The integration profile of EIAV-based vectors. Mol. Ther..

[91] Trobridge G.D., Miller D.G., Jacobs M.A., Allen J.M., Kiem P., Kaul R., Russell D.W. (2006). Foamy virus vector integration sites in normal human cells. Proc. Natl. Acad. Sci. USA.

[92] Nowrouzi A., Dittrich M., Klanke C., Heinkelein M., Rammling M., Dandekar T., von Kalle C., Rethwilm A. (2006). Genome-wide mapping of foamy virus vector integrations into a human cell line. J. Gen. Virol..

[93] Kawasuji T., Fuji M., Yoshinaga T., Sato A., Fujiwara T., Kiyama R. (2006). A platform for designing HIV integrase inhibitors. Part 2: A two-metal binding model as a potential mechanism of HIV integrase inhibitors. Bioorg. Med. Chem..

[94] Andrake M.D., Skalka A.M. (1996). Retroviral integrase, putting the pieces together. J. Biol. Chem..

[95] Bujacz G., Alexandratos J., Wlodawer A., Merkel G., Andrake M., Katz R.A., Skalka A.M. (1997). Binding of different divalent cations to the active site of avian sarcoma virus integrase and their effects on enzymatic activity. J. Biol. Chem..

[96] Chen J.C., Krucinski J., Miercke L.J., Finer-Moore J.S., Tang A.H., Leavitt A.D., Stroud R.M. (2000). Crystal structure of the HIV-1 integrase catalytic core and C-terminal domains: A model for viral DNA binding. Proc. Natl. Acad. Sci. USA.

[97] Jaskolski M., Alexandratos J.N., Bujacz G., Wlodawer A. (2009). Piecing together the structure of retroviral integrase, an important target in AIDS therapy. FEBS J..

[98] Mitsuya H., Weinhold K.J., Furman P.A., St Clair M.H., Lehman S.N., Gallo R.C., Bolognesi D., Barry D.W., Broder S. (1985). 3'-Azido-3'-deoxythymidine (BW A509U): An antiviral agent that inhibits the infectivity and cytopathic effect of human T-lymphotropic virus type III/lymphadenopathy-associated virus *in vitro*. Proc. Natl. Acad. Sci. USA.

[99] De Clercq E. (2009). The history of antiretrovirals: Key discoveries over the past 25 years. Rev. Med. Virol..

[100] Marchand C. (2012). The elvitegravir Quad pill: The first once-daily dual-target anti-HIV tablet. Expert Opin. Investig. Drugs.

[101] Murray J.M., Emery S., Kelleher A.D., Law M., Chen J., Hazuda D.J., Nguyen B.Y., Teppler H., Cooper D.A. (2007). Antiretroviral therapy with the integrase inhibitor raltegravir alters decay kinetics of HIV, significantly reducing the second phase. AIDS.

[102] Shimura K., Kodama E., Sakagami Y., Matsuzaki Y., Watanabe W., Yamataka K., Watanabe Y., Ohata Y., Doi S., Sato M. (2008). Broad antiretroviral activity and resistance profile of the novel human immunodeficiency virus integrase inhibitor elvitegravir (JTK-303/GS-9137). J. Virol..

[103] Zolopa A.R., Berger D.S., Lampiris H., Zhong L., Chuck S.L., Enejosa J.V., Kearney B.P., Cheng A.K. (2010). Activity of elvitegravir, a once-daily integrase inhibitor, against resistant HIV Type 1: Results of a phase 2, randomized, controlled, dose-ranging clinical trial. J. Infect. Dis..

[104] (2012). FDA approves new 4-drug once-a-day HIV treatment. AIDS Policy Law..

[105] Rabaaoui S., Zouhiri F., Lancon A., Leh H., d’Angelo J., Wattel E. (2008). Inhibitors of strand transfer that prevent integration and inhibit human T-cell leukemia virus type 1 early replication. Antimicrob. Agents Chemother..

[106] Smith R.A., Gottlieb G.S., Miller A.D. (2010). Susceptibility of the human retrovirus XMRV to antiretroviral inhibitors. Retrovirology.

[107] Wei S.Q., Mizuuchi K., Craigie R. (1997). A large nucleoprotein assembly at the ends of the viral DNA mediates retroviral DNA integration. EMBO J..

[108] Hearps A.C., Jans D.A. (2006). HIV-1 integrase is capable of targeting DNA to the nucleus via an importin alpha/betadependent mechanism. Biochem. J..

[109] Ao Z., Fowke K.R., Cohen E.A., Yao X. (2005). Contribution of the C-terminal tri-lysine regions of human immunodeficiency virus type 1 integrase for efficient reverse transcription and viral DNA nuclear import. Retrovirology.

[110] Levin A., Armon-Omer A., Rosenbluh J., Melamed-Book N., Graessmann A., Waigmann E., Loyter A. (2009). Inhibition of HIV-1 integrase nuclear import and replication by a peptide bearing integrase putative nuclear localization signal. Retrovirology.

[111] Ao Z., Huang G., Yao H., Xu Z., Labine M., Cochrane A.W., Yao X. (2007). Interaction of human immunodeficiency virus type 1 integrase with cellular nuclear import receptor importin 7 and its impact on viral replication. J. Biol. Chem..

[112] Zaitseva L., Cherepanov P., Leyens L., Wilson S.J., Asaiyaah J., Fassati A. (2009). HIV-1 exploits importin 7 to maximize nuclear import of its DNA genome. Retrovirology.

[113] Christ F., Thys W., de Rijck J., Gijsbers R., Albanese A., Arosio D., Emiliani S., Rain J.C., Benarous R., Cereseto A. (2008). Transportin-SR2 imports HIV into the nucleus. Curr. Biol..

[114] Luban J. (2008). HIV-1 infection: Going nuclear with TNPO3/Transportin-SR2 and integrase. Curr. Biol..

[115] Rain J.C., Cribier A., Gerard A., Emiliani S., Benarous R. (2009). Yeast two-hybrid detection of integrase-host factor interactions. Methods.

[116] Zennou V., Petit C., Guetard D., Nerhbass U., Montagnier L., Charneau P. (2000). HIV-1 genome nuclear import is mediated by a central DNA flap. Cell.

[117] Llano M., Vanegas M., Fregoso O., Saenz D., Chung S., Peretz M., Poeschla E.M. (2004). LEDGF/p75 determines cellular trafficking of diverse lentiviral but not murine oncoretroviral integrase proteins and is a component of functional lentiviral preintegration complexes. J. Virol..

[118] Cherepanov P., Maertens G., Proost P., Devreese B., Van Beeumen J., Engelborghs Y., De Clercq E., Debyser J. (2003). HIV-1 integrase forms stable tetramers and associates with LEDGF/p75 protein in human cells. J. Biol. Chem..

[119] Kataoka N., Bachorik J.L., Dreyfuss G. (1999). Transportin-SR, a nuclear import receptor for SR proteins. J. Cell Biol..

[120] Lai M.C., Lin R.I., Huang S.Y., Tsai C.W., Tarn W.Y. (2000). A human importin-beta family protein, transportin-SR2, interacts with the phosphorylated RS domain of SR proteins. J. Biol. Chem..

[121] Levin A., Hayouka Z., Friedler A., Loyter A. (2010). Transportin 3 and importin a are required for effective nuclear import of HIV-1 integrase in virus-infected cells. Nucelus.

[122] Craigie R. (2001). HIV integrase, a brief overview from chemistry to therapeutics. J. Biol. Chem..

[123] Peters K., Wiktorowicz T., Heinkelein M., Rethwilm A. (2005). RNA and protein requirements for incorporation of the Pol protein into foamy virus particles. J. Virol..

[124] Bouyac-Bertoia M., Dvorin J.D., Fouchier R.A., Jenkins Y., Meyer B.E., Wu L.I., Emerman M., Malim M.H. (2001). HIV-1 infection requires a functional integrase NLS. Mol Cell.

[125] Kukolj G., Jones K.S., Skalka A.M. (1997). Subcellular localization of avian sarcoma virus and human immunode ficiency virus type 1 integrases. J. Virol..

[126] Bukrinsky M.I., Haggerty S., Dempsey M.P., Sharova N., Adzhubel A., Spitz L., Lewis P., Goldfarb D., Emerman M., Stevenson M. (1993). A nuclear localization signal within HIV-1 matrix protein that governs infection of non-dividing cells. Nature.

[127] Connor R.I., Chen B.K., Choe S., Landau N.R. (1995). Vpr is required for efficient replication of human immunodeficiency virus type 1 in mononuclear phagocytes. Virology.

[128] Heinzinger N.K., Bukinsky M.I., Haggerty S.A., Ragland A.M., Kewalramani V., Lee M.A., Gendelman H.E., Ratner L., Stevenson M., Emerman M. (1994). The Vpr protein of human immunodeficiency virus type 1 influences nuclear localization of viral nucleic acids in nondividing host cells. Proc. Natl. Acad. Sci. USA.

[129] An D.G., Hyun U., Shin C.G. (2008). Characterization of nuclear localization signals of the prototype foamy virus integrase. J. Gen. Virol..

[130] Mullers E., Stirnnagel K., Kaulfuss S., Lindemann D. (2011). Prototype foamy virus gag nuclear localization: A novel pathway among retroviruses. J. Virol..

[131] Tobaly-Tapiero J., Bittoun P., Lehmann-Che J., Delelis O., Giron M.L., de Thé H., Saïb A. (2008). Chromatin tethering of incoming foamy virus by the structural Gag protein. Traffic.

[132] Lo Y.T., Tian T., Nadeau P.E., Park J., Mergia A. (2010). The foamy virus genome remains unintegrated in the nuclei of G1/S phase-arrested cells, and integrase is critical for preintegration complex transport into the nucleus. J. Virol..

